# Intrinsic Affective Network Is Impaired in Children with Attention-Deficit/Hyperactivity Disorder

**DOI:** 10.1371/journal.pone.0139018

**Published:** 2015-09-25

**Authors:** New-Fei Ho, Joanna S. X. Chong, Hui Li Koh, Eleni Koukouna, Tih-Shih Lee, Daniel Fung, Choon Guan Lim, Juan Zhou

**Affiliations:** 1 Center for Cognitive Neuroscience, Neuroscience & Behavioral Disorders Program, Duke-National University of Singapore Graduate Medical School, Singapore, Singapore; 2 Research Division, Institute of Mental Health, Singapore, Singapore; 3 Department of Child and Adolescent Psychiatry, Institute of Mental Health, Singapore, Singapore; 4 Clinical Imaging Research Centre, The Agency for Science, Technology and Research-National University of Singapore, Singapore, Singapore; Central Institute of Mental Health, GERMANY

## Abstract

Deficits in impulsivity and affect dysregulation are key features of attention-deficit/hyperactivity disorder (ADHD) besides impairing levels of hyperactivity and/or inattention. However, the neural substrates underlying these traits are relatively under-investigated. In this study, we use resting-state functional magnetic resonance imaging to test the hypothesis of diminished functional integration within the affective/limbic network (which includes the amygdala, hippocampus, subgenual cingulate cortex, orbitofrontal cortex and nucleus accumbens) of children with ADHD, which is associated with their behavioral measures of emotional control deficits. Resting state-fMRI data were obtained from 12 healthy control subjects and 15 children with ADHD, all who had a minimum one-month washout period for medications and supplements. Children with ADHD demonstrated less integrated affective network, evidenced by increased bilateral amygdalar and decreased left orbitofrontal connectivity within the affective network compared to healthy controls. The hyper-connectivity at the left amygdalar within the affective network was associated with increased aggressiveness and conduct problems, as well as decline in functioning in children with ADHD. Similar findings in affective network dysconnectivity were replicated in a subset of children with ADHD three months later. Our findings of divergent changes in amygdala and orbitofrontal intrinsic connectivity support the hypothesis of an impaired functional integration within the affective network in childhood ADHD. Larger prospective studies of the intrinsic affective network in ADHD are required, which may provide further insight on the biological mechanisms of emotional control deficits observed in ADHD.

## Introduction

Affect dysregulation is a key feature of attention-deficit/hyperactivity disorder (ADHD) in children [1,2]. Although not classified among the core symptoms of impairing and developmentally inappropriate levels of hyperactivity, impulsivity and/or inattention in more recent versions of the Diagnostic and Statistical Manual of Mental Disorders, deviations in affect were previously considered a primary clinical symptom in earlier classifications of childhood ADHD [3]. ADHD children with affect dysregulation often display uncontrolled outbursts of emotion, rapid and exaggerated changes in mood (emotional lability), and are easily frustrated with unexpected emotional challenges [2,4]. Also, affect dysregulation in childhood has been associated with subsequent psychopathology, poor psychosocial functioning and quality of life in ADHD well into adulthood [4–8].

Abnormalities in large-scale, distributed brain networks are now widely thought to underlie the core features of mental disorders, including ADHD [9,10]. An increasingly popular approach to measure whole-brain networks is through the use of resting-state functional magnetic resonance imaging (RS-fMRI) [9,10]. RS-fMRI methods examine the temporal correlations between the low-frequency oscillations in blood-oxygenation-level-dependent (BOLD) signals in different regions of the brain when subjects are lying quietly in the scanner without doing a task [11,12]. Many large-scale networks have been reliably identified using RS-fMRI methods in different populations across multiple investigator sites [13–15]. Measures of RS-fMRI brain networks or otherwise known as intrinsic connectivity networks [16] have been shown to be sensitive to the effects of disease and ageing [11,17]. The use of RS-fMRI to study brain networks in the young population, especially a clinical cohort like ADHD, is well suited (Uddin, 2010). Compared to task-based scans, resting-state scans are independent of cognitive demands and take a considerable shorter amount of time to collect [18].

Two distinct methodological approaches are widely used to analyze RS-fMRI measures. The first approach is region-of-interest (ROI) or “seed”-based. It is a hypothesis-driven as one or two ROI/seeds are chosen *a priori*. The RS-fMRI measures of other regions, typically whole brain, are then correlated with the seed RS-fMRI measures to obtain a functional connectivity map of the seed [11,19]. The second approach is independent component analysis (ICA), a statistical method that separates signals of no interest (such as those from the cerebrospinal fluid in the ventricles and the white matter, and those from motion) from the signals of interest (the low frequency spatially-distributed synchronization) [20]. It is data-driven as no prior information about spatial location, size and shape of a focal region-of-interest is required [13,20]. The seed-based and ICA approaches have been applied in several studies of childhood ADHD [21–24]. Other methods to analyze RS-fMRI in childhood ADHD include using graph theory [25] and amplitude of low frequency fluctuations [24].

Emerging RS-fMRI studies of ADHD have shown abnormalities in large-scale resting-state networks such as the frontoparietal, dorsal attention and default mode networks, which are associated with ADHD-related behaviors of impaired executive control, lack of attention, and mind-wandering [9,18,21,23,24,26–29]. However, the role of the resting-state affective network in ADHD remains relatively under-investigated. One of the earliest brain networks identified, the affective or limbic network has long been regarded as the “emotional brain” [30–32]. Both animal and human neuroimaging studies have shown the integral role of the affective network in emotion-based decision making, reward and motivation [33,34]. Key components of the affective network include hippocampus, parahippocampal gyrus, amygdala, orbitofrontal cortex, medial frontal cortex, nucleus accumbens (ventral striatum), subgenual anterior cingulate cortex and anterior insula. Task-based fMRI studies have shown impaired BOLD activation in key components of the affective network in subjects with ADHD. For instance, amygdalar hyperactivation, ventral striatum hypoactivation and orbitofrontal hyperactivation have been found during many emotional perception and reward processing tasks [1,28,35–38]. The abnormal BOLD activity elicited during these emotional task states suggests that the intrinsic affective networks in subjects with ADHD may be different from healthy subjects.

Three recent seed-based RS-fMRI studies of ADHD—one conducted in the adult cohort and the other two in the child cohort, have examined components of the intrinsic affective network [39,40]. Hyperconnectivity of the superior parietal lobe and cerebellum to the anterior cingulate cortex was found in adults diagnosed with ADHD in childhood compared with healthy comparison adults [39]. Affective network hypoconnectivity, however, was found in medication naïve children with ADHD compared with healthy comparison children [40]. Specifically, there was decreased connectivity between the following affective network regions: orbitofrontal cortex, hippocampus, and anterior prefrontal cortex, with the ventral striatum in children with ADHD [40]. The third study found hyperconnectivity between the amygdala and rostral anterior cingulate cortex in a subpopulation of ADHD children with high emotional lability [22]. The divergent findings of both hyperconnectivity and hypoconnectivity within the affective network, although shown by using different seeds, suggest that the intrinsic affective connectivity network in ADHD could be less integrated or even disorganized.

In this study, we hypothesized a divergent, less integrated affective network (especially between the frontal and amygdalar regions) in children with ADHD, as compared to healthy controls. We predicted that the altered affective functional connectivity in ADHD is associated with behavioral measures of emotional control dysregulation. To complement the prior seed-based findings of the affective network in ADHD, we adopted a data-driven approach (ICA) to measure the functional connectivity within the affective network. This approach is independent of the seed definition and automatically removes motion and physiological noise [20]. The spatial pattern of the affective network was based on one that has been identified in 500 healthy adults and replicated with an independent cohort of 500 subjects [9,41]. We compared the intrinsic affective connectivity network between children with ADHD and healthy controls. We also measured the intrinsic affectivity network in a subset of children with ADHD three months later to investigate whether the group-based differences are enduring. In the overall ADHD cohort, as well as the hyperactive-impulsive/inattention subtype of ADHD subjects, we correlated the functional connectivity in affective regions exhibiting group-based differences with clinical measures of affect control.

## Materials and Methods

### Participants

The investigation was carried out in accordance with the guidelines of the Institutional Review Board for the National Healthcare Group, the Singapore Health Services Group and National University of Singapore. Written informed consent from the parents and assent forms from the child to partake in the studies and to allow imaging data for further analyses were both obtained after the nature of the procedures had been fully explained.

19 children with ADHD were recruited at the Child Guidance Clinic, Institute of Mental Health. The subjects with ADHD were diagnosed by child psychiatrists according to the Diagnostic and Statistical Manual-Fourth Edition (DSM-IV) for ADHD. Additionally, parents were also interviewed using the Diagnostic Interview Schedule for Children (DISC), which is based on DSM-IV. The subjects with ADHD were either the inattentive or combined subtypes. 3 of the subjects with ADHD had a comorbidity of either developmental dyslexia and/or developmental reading disorder. Prior to the study, 3 subjects with ADHD were on methylphenidate and 5 subjects were taking fatty acid supplementation. They were only allowed to participate in study procedures after at least one month of washout. The rest of the subjects were medication naive. MRI scans from a subset of the subjects with ADHD (11 out of 19) were additionally collected three months later. Throughout this period, these follow-up subjects remained medication and supplementation free. 16 demographically-matched healthy controls were recruited via advertisements from both the Child Guidance Clinic and National University of Singapore. The controls had no present or history of mental disorders. Exclusion criteria for all subjects included history of epileptic seizures, mental retardation and an IQ of less than 70.

### Neuropsychological assessments

Neuropsychological assessments were administered on children with ADHD at baseline. No assessments were performed on healthy subjects or the children with ADHD at follow-up. The rating instruments used in this study are the Child Behavior Checklist-Parents (CBCL) [42] and Children Global Assessment Scale [43]. The CBCL is a parent-rated questionnaire designed to obtain descriptions of a child’s competencies and behavioral/emotional problems and consists of 118 items [44]. It provides both empirical-based symptoms and dimensional constructs for psychopathology, and is well validated [45]. Scores for 8 syndrome scales are derived from factor analyses of the CBCL. In addition, 6 DSM-5-oriented scales are also derived from the CBCL[44]. For this study, 4 syndrome scales (Aggression, Rule-breaking behavior, Affective problems, Anxiety-depression problems) were chosen as measures of dysregulated mood; these scales have been used in the scoring of aggression in ADHD, as well as anxiety-depression and affective problems in atypical developing children [46–48]. As dysregulated affect is also a core feature of clinically-overlapping disorders of oppositional defiant and conduct [49], the dimensional constructs of oppositional defiant and conduct problems were also selected for subsequent brain-behavior correlations.

Additionally, as a marker of deficient emotional self-regulation (CBCL-DESR) in ADHD [50], t-scores from three items on the CBCL were summed, namely Anxiety/depression, Aggression and Attention to reflect intense emotions, aggression and impulsive behavior respectively. Separately, the Children Global Assessment Scale [43] was used to determine the child’s level of general functioning at home, school or with peers.

### Image acquisition

All functional and structural images were collected at the Duke-National University of Singapore Graduate Medical School as part of a multi-modal imaging protocol using a 12-channel phase-array head coil on a 3-Tesla Tim Trio scanner (Siemens, Germany). The RS-fMRI data using T2*-weighted echo planar images (repetition time = 2000 ms, echo time = 30 ms, flip angle = 90 degrees, field of view = 192 x 192 mm^2^, voxel size = 3.0 mm isotropic, slice thickness = 3 mm, no gap, 36 axial slices, interleaved collection) were collected while the subjects were asked to relax and stare at a cross centered on a screen. The RS-fMRI data collection (8 minutes 12 seconds altogether; 246 volumes) was broken up into two consecutive short runs to minimize motion artifacts; the duration for each of the two runs were 4 min 6 seconds each. An eye tracker was used to ensure that the children stayed awake for the entire RS-fMRI scan. The high-resolution structural T1-weighted magnetization prepared rapid gradient echo images (repetition time = 2300 ms, echo time = 2.98 ms, inversion time = 900 ms, flip angle = 90 degrees, field of view = 256 x 256mm^2^, voxel size = 1.0 mm isotropic) were collected for atlas registration of the RS-fMRI images, as well as for subsequent structural analysis of voxel-based morphometry (VBM). After three months, an additional set of the same scans was collected from a subset of children with ADHD on the same scanner.

### Image preprocessing

The task-free fMRI data were preprocessed using procedures outlined in a previous study [51] using the FMRIB Software Library (FSL) [52] and the Analysis of Functional NeuroImages software program [53]. The preprocessing included the following steps: 1) discarding the first 6 volumes that were collected while the magnetic field has not yet stabilized, 2) correction for the intensity differences between odd and even volumes during the interleaved volume acquisition, 3) motion correction, 4) skull stripping, 5) spatial smoothing using a 6 mm full width half maximum (FWHM) Gaussian kernel to improve signal-to-noise ratio and to reduce inter-subject variability, and 6) co-registering to the structural MRI image using Boundary-Based Registration [54] and then registering the image to the Montreal Neurological Institute (MNI) 152 standard space of 2 mm isotropic resolution using a nonlinear registration tool (FNIRT). The preprocessed data of both runs were concatenated for further analyses.

As excessive motion can introduce spurious functional correlations in intrinsic networks [55,56], high-motion frames for each individual were removed. These high-motion frames were identified through inter-frame motion parameters and intensity differences calculated during the pre-processing steps [55]. We removed frames with more than 0.5 framewise displacement (FD) and an inter-frame signal intensity difference [55] of more than 0.065 from each subject for analysis; the cut-off for the maximum number of frames that could be removed from each subject was less than 10%. For 4 of the initial 19 subjects with ADHD, 1 of the 11 subjects with ADHD with a second scan, and 4 of the initial 16 healthy controls, their entire data were excluded from analyses as they moved excessively throughout the entire scan (i.e. percentage of high-motion frames were more than 10% of the entire set of RS-fMRI frames). Hence, the tally for subsequent analysis of MRI data was 12 healthy children volunteers, 15 ADHD children with baseline data, and 10 ADHD children (out of the 15) with follow-up data.

The structural T1-weighted MRI data of all subjects passed the visual check for motion artifacts. VBM was performed for each subject using SPM8 (Wellcome trust centre for neuroimaging, http://www.fil.ion.ucl.ac.uk/spm/software/spm8/) [57],. Grey matter volume was obtained using the following preprocessing steps 1) segmentation of individual structural MRI into cerebral spinal fluid, grey and white matter, 2) normalization of the resulting grey matter maps into the standard MNI space using a Diffeomorphic Anatomical Registration Through Exponentiated Lie Algebra (DARTEL) procedure, 3) nonlinear modulation of grey matter maps to compensate for spatial normalization effects, and 4) smoothing of the modulated images with a isotropic Gaussian kernel with 8mm FWHM [58]. The resulting grey matter probabilistic maps were used for atrophy correction.

### Intrinsic network extraction

The pre-processed the RS-fMRI signals were decomponsed into spatially independent components using open-source Group ICA Toolbox (GIFT, Medical Image Analysis Lab; http://mialab.mrn.org/software/). To extract the spatial pattern of the affective network from each individual, the following steps were performed: 1) concatenating the preprocessed data of 2 runs of 4 min 6s for each subject across groups, 2) combining the data across all groups (including healthy controls and ADHD subjects at baseline, as well as follow-up data from the ADHD subjects) into an aggregate dataset, 2) using principal component analysis to perform initial reduction of random variables, 3) extracting thirty group-level independent components using minimum description length criteria, with reference to previous studies [59,60], 4) visually identifying the group-level independent component corresponding to the affective/limbic network with reference to previous published large-scale network templates identified based on 500 healthy adults and replicated with an independent cohort of 500 subjects [41], 5) back-projecting the affective network-related independent component from the aggregate dataset into each individual to yield individual-specific spatial maps of the affective network at baseline and follow-up [61].

### Statistical analysis

Demographic and clinical differences between the groups (dataset 1: 15 subjects with ADHD and 10 healthy controls, and dataset 2: 10 subjects with ADHD that underwent additional MRI scans three months later and the same 10 healthy controls) were tested using either two-tailed independent sample t-tests (age, number of quality RS-fMRI frames used for analysis, parameters for motion displacement) or chi-squared tests (gender, ethnicity and handedness).

To examine the validity of the affective networks identified in both datasets, group-level average functional connectivity map of the affective network were created using one-sample t-test across the individual spatial maps of the affective network (both controls and ADHD) [62], regressing out for the individual effects of age, gender, number of fMRI frames used in the analysis, and mean maximal absolute signal displacement across the chosen frames, corrected at family-wise error (FWE) rate of *p* < .05. To compare the affective network connectivity between the subjects with ADHD and healthy subjects, two-sample t-tests with the same regressors were performed. Following our previous work [63], we identified significant clusters using a joint height (p<0.01) and extent (p<0.05, corresponding to a minimum cluster size of 62 voxels as computed by SPM) probability threshold, corrected at whole-brain level [64]. The statistical maps were masked explicitly to the relevant affective network by binarizing the two-group average network map at a height threshold of p<0.001 (uncorrected). The Anatomical Automatic Labeling toolbox (http://www.gin.cnrs.fr/) was used to identify the specific brain region showing group-wise functional connectivity differences within the affective network. The MarsBar toolbox (http://marsbar.sourceforge.net/) was used to extract the subject-level mean affective network connectivity in the above regions with group-wise differences.

To examine whether the group differences in functional connectivity of the affective network could be explained by the underlying grey matter volume, we performed the voxelwise grey matter volume correction following our previous method [63]. The Biological Parametric Mapping (BPM) toolbox [65] was used to incorporate the subject-level voxelwise grey matter volume probabilistic map derived by VBM, as well as age, gender, number of fMRI frames used in the analysis, and mean maximal absolute signal displacement across the chosen frames as covariates. The same thresholding was applied to determine group differences in functional connectivity.

To examine whether there was a relationship between behavioral constructs of affect problems in the 15 subjects with ADHD and the region displaying group-wise differences, post-hoc Pearson correlational analysis was performed on the six CBCL subscales. Multiple comparisons were performed using the Bonferroni-Holm method to control for Type 1 errors at a FWE *p* < .05. Additional clinical correlations were performed with scores on the CBCL-DESR, as well as the Child Global Assessment Scale. Furthermore, we conducted similar post-hoc correlational analyses on 10 subjects with the combined subtype of ADHD (hyperactive-impulsive and inattention), excluding 5 inattentive subtype ADHD subjects.

## Results

**Fig 1:**
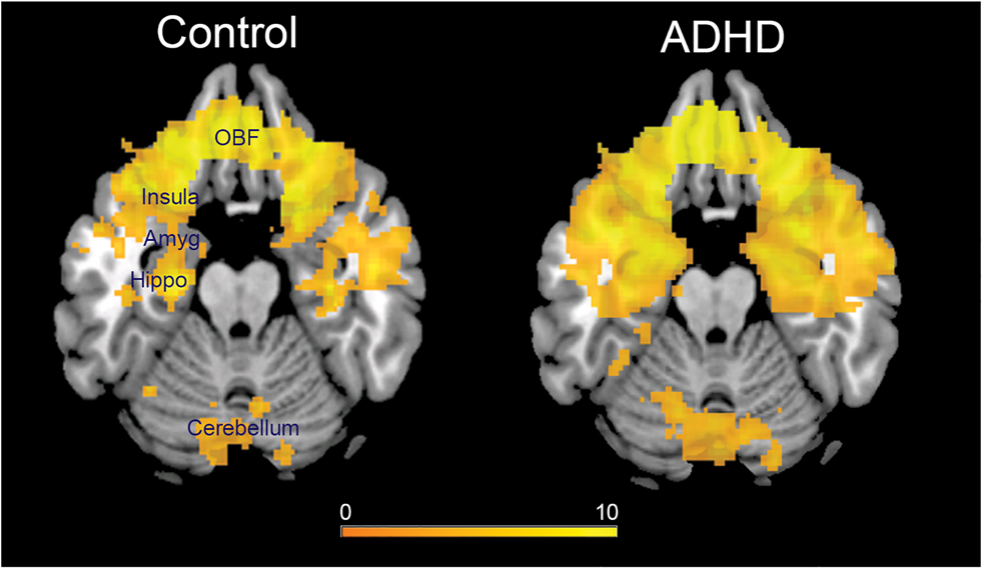
Affective networks in healthy control and ADHD groups identified using independent component analysis (ICA). Resting-state data from all the subjects (healthy control children and children with ADHD) were aggregated into group-level ICA. The combined affective network across all the subjects was identified by visual spatial template matching with a previously identified affective/limbic network of 1000 subjects [41]. Individual maps of the affective network were then constructed by back-projection. The group-level average affective network for healthy control children and ADHD children are presented here at p<0.05 FWE corrected. Color bar represents t-statistics.

There were no significant differences in the demographic variables and imaging variables (including motion and number of RS-fMRI volumes used for analyses) between the ADHD cohort and healthy controls in either dataset 1 (**Table 1)** or dataset 2 (**Table 2**). The brain structures of the affective network identified by ICA were namely the amygdala, anterior hippocampus, parahippocampal gyrus, basal forebrain (subsuming nucleus accumbens and hypothalamus), subgenual anterior cingulate cortex, ventromedial prefrontal cortex subsuming the orbitofrontal cortex, anterior insula and cerebellum (**Fig 1**).

**Table 1:** Demographic, imaging and clinical information of participants in dataset 1.

	Healthy (N = 12)	ADHD (N = 15)	*t* or χ^2^ value	*p*-value
Gender	10 males	15 males	2.7	0.19
Age (years)	10.30 (2.31)	9.40 (1.24)	-1.35	0.19
Handedness	11 right	15 right	1.3	0.44
Ethnicity	12 Chinese	14 Chinese, 1 Indian	0.83	1
Number of good fMRI volumes	199.25 (43.73)	212.67 (26.69)	0.93	0.36
Percentage of good fMRI volumes (%)	92.52 (14.40)	93.73 (8.17)	0.28	0.78
Absolute motion displacement, mm	1.01 (0.88)	1.02 (0.90)	0.03	0.98
ADHD Combined subtype (N)	-	10		
ADHD Inattentive subtype (N)	-	5		
Age onset of disorder (years)	-	5.0 (0.93)		
CBCL-DESR scores	-	193.87 (15.78)		
Children Global Assessment Scale	-	58.13 (5.45)		

Descriptive statistics of healthy participants and ADHD participants at the first scan. Continuous variables are expressed as mean (standard deviation). N: number of subjects. CBCL-DESR: Child Behavioral Checklist (Parent)—Deficit in Emotional Self-Regulation.

**Table 2:** Demographic, imaging and clinical information of participants in dataset 2.

	Healthy (N = 12)	ADHD-2 (N = 10)	*t* or χ^2^ value	*p*-value
Gender	10 males	10 males	1.83	0.48
Age	10.30 (2.3)	9.60 (1.5)	-0.86	0.4
Handedness	11 right	10 right	0.87	1
Ethnicity	12 Chinese	9 Chinese, 1 Indian	1.26	0.46
Number of good fMRI volumes	199.25 (43.73)	210.40 (28.71)	0.72	0.48
Percentage of good fMRI volumes (%)	92.52 (14.40)	91.85 (9.50)	-0.13	0.9
Absolute motion displacement, mm	1.01 (0.88)	1.27 (0.99)	0.64	0.53
ADHD Combined subtype (N)	-	6		
ADHD Inattentive subtype (N)	-	4		
Age onset of disorder (years)	-	5.10 (0.88)		

Descriptive statistics of the healthy participants (at the baseline scan) and the subgroup of ADHD participants at the follow-up scan. 10 out of 15 ADHD subjects scanned at baseline were scanned again after three months. Continuous variables are expressed as mean (standard deviation). N = number of subjects

### Increased amygdalar connectivity and decreased orbitofrontal connectivity in childhood ADHD

**Fig 2:**
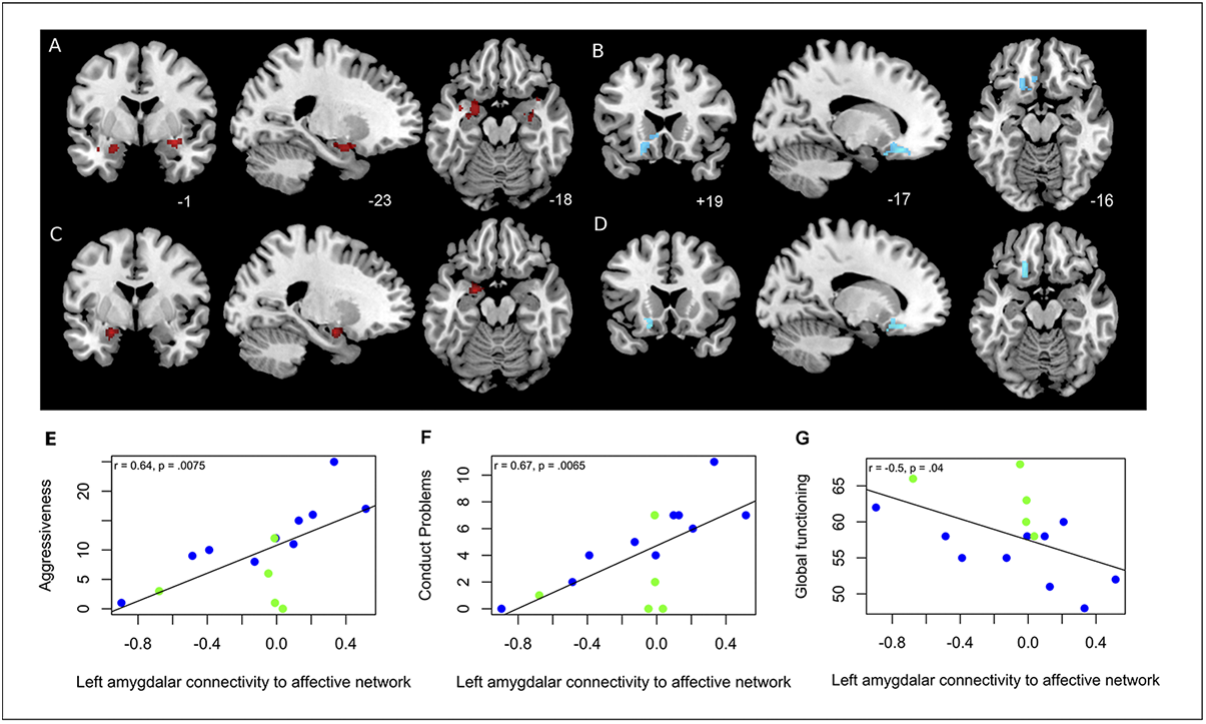
Disrupted affective network connectivity in ADHD compared to healthy controls. Group comparisons of the affective network at baseline (dataset 1) show increased left amygdalar connectivity (A) and decreased left orbitofrontal cortex connectivity (B) in children with ADHD compared to healthy control children. Similar significant group-wise findings were observed in a subset of 10 ADHD children three months later compared to healthy subjects (C, D). E-G brain-behavior scatterplots: The increased left amygdalar connectivity in ADHD children correlated with increased scores of aggressiveness (E) and conduct problems (F). The correlation between left increased amygdalar connectivity and lower general functioning is also depicted (G). Children with the ADHD combined subtype (both inattention and impulsive) are labeled blue while children with ADHD inattention subtype are labeled green.

Compared to healthy subjects, children with ADHD in dataset 1 displayed greater left and right amygdala (left: -24, -4, -18; cluster of 169 voxels (p = .007), peak z-score of 3.20; and right: 28, -1, -13; cluster of 177 voxels (p = .009), peak z-score of 3.68) and decreased left orbitofrontal (-16, 20, -17; cluster of 218 voxels (p = .006), peak z-score of 3.98) connectivity within the affective network (**Fig 2A & 2B**). The findings of increased left amygdalar (-22, -2, -18; clusters of 87 voxels, peak z-score of 4.41) and marginally decreased left orbitofrontal (-18, 18, -16; cluster of 60 voxels, peak z-score of 3.22) connectivity were replicated in a subset of ADHD subjects three months later (**Fig 2C & 2D**).

### Abnormalities in intrinsic affective network connectivity in childhood ADHD could not be explained by grey matter volume

When intra-subject variability in grey matter anatomy was accounted for, the group-based functional connectivity differences remained intact. i.e. the size and magnitude of the cluster either increased or were similar (left amygdala: cluster of 162 voxels (p = .006), peak z-score of 3.15; right amygdala: cluster of 160 voxels (p = .005), peak z-score of 4.03; and left orbitofrontal cortex: cluster of 216 voxels (p = .002), peak z-score of 3.94). The same was observed for the subset of ADHD after three months.

### Anomalies within the intrinsic affective network are associated with dysregulated emotional control in childhood ADHD

Post-hoc correlations in dataset 1 revealed that the increase in left amygdalar connectivity significantly correlated with measures of conduct problems (r = 0.67, p = .0065) (Fig 2E) and aggressiveness (r = 0.64, p = .0075) after multiple comparisons (FWE rate p < .05). Positive trends in association were also noted for the other measures of emotional control (rule-breaking: r = 0.62, oppositional defiant problems: r = 0.62, anxiety problems: r = 0.36, affective problems: r = 0.35). An inverse correlation between increased amygdalar connectivity and lower global functioning was also found (r = -0.5, p = .04) (Fig 2F).

### Secondary analysis on ADHD combined subtypes

Post-hoc analysis of ten children with ADHD with combined subtypes (i.e. excluding five subjects with inattention subtypes) compared to healthy controls, revealed enhanced significant associations between increased amygdalar connectivity and increased emotional control problems (rule-breaking: r = 0.91, p = .0002; conduct problems: r = 0.88, p = .0009; aggressiveness: r = 0.86, p = .0016 and oppositional defiant problems: r = 0.85, p = .0017) after multiple comparisons. Significant positive association with the CBCL-DESR (r = 0.67, p = .033) and negative association with the global function (r = -0.65, p = .03) were also found in children with ADHD-combined subtype.

## Discussion

We present evidence of abnormal affective network connectivity in children with ADHD. Compared to healthy controls, children with ADHD displayed reduced orbitofrontal connectivity and heightened amygdalar connectivity within the affective network. Critically, these intrinsic connectivity changes in ADHD remained after three months. The heightened amygdalar connectivity in children with ADHD significantly correlated with emotional dysregulation problems and a decline in general functioning. Our findings suggest a less integrated affective intrinsic network in children with ADHD that might underlie their emotional dysfunction.

### Impaired affective intrinsic connectivity in childhood ADHD

Previous seed-based studies of the affective network have found mixed results of hyper-connected and hypo-connected affective network in ADHD [22,39,40]. Our findings, based on a data-driven approach to extract the entire affective network, also indicate the phenomenon of hyperconnectivity and hypoconnectivity within the affective network. This shows that that previous findings are not irreconcilable and taken together, points to a divergent intrinsic affective network (particularly between frontal and amygdala) in childhood ADHD.

The abnormal affective networks in ADHD children may underlie parts of their behavioral problems. One study has shown that the hypoconnectivity between the left orbitofrontal cortex and left ventral striatum of the affective network correlated with the emotional lability measures in medication naïve children with ADHD [40]. Another study has shown that the functional connectivity between the amygdala and subgenual anterior cingulate cortex positively correlated with emotional lability measures in ADHD children that were classified with high emotional lability [22]. Similarly, our findings of amygdalar hyperconnectivity within the affective network were associated with behavioral constructs of affect dysregulation, including rule-breaking and aggression. When we narrowed the analysis to the children diagnosed with combined subtypes of ADHD, the relationship between amygdalar hyperconnectivity and measures of emotional control deficits, including a neuropsychological marker for deficits in emotional self-regulation became stronger. However, as the sample size of our ADHD study participants is small, a caveat is that the observed significant brain-behavioral correlations may not be that reliable [66].

### Abnormal intrinsic affective connectivity in children with ADHD is not due to grey matter variability

Till date, the neurobiological substrates of intrinsic functional connectivity are not yet known. Although structural deficits in grey matter of the limbic regions in children and adults with ADHD have been previously reported [67,68], our findings indicate that the altered intrinsic affective connectivity in children with ADHD is not dictated by individual grey matter variability.

Non-human primate evidence suggests that intrinsic functional connectivity correspond to direct and indirect anatomical connections [69]. By using diffusion tensor imaging measures of structural connectivity—indices of white matter microstructure—abnormalities in the frontal-accumbens structural connectivity in children with ADHD have recently been observed [46]. More specifically, the decreased structural connectivity between the left nucleus accumbens and left orbitofrontal cortex were associated with increased aggression [46] Another index of white matter integrity: fractional anisotropy has been found to be higher in the temporal regions but lower in the orbitofrontal cortex in medication naïve adults with ADHD compared to healthy controls [10]. The fractional anisotropy measures of orbitofrontal fiber tracts inversely correlated with measures of impulsivity in ADHD [10]. We have also previously reported white matter alterations in a follow-up study of adults with ADHD [70]. Hence, it is possible that the altered connectivity within the intrinsic affective network corresponds to, or may be influenced by possible alterations in white matter integrity.

### Abnormal intrinsic affective connectivity parallel patterns of abnormal emotional task-based activity

The divergent patterns of hypoconnectivity of the orbitofrontal cortex and hyperconnectivity of the amygdala in our findings correspond to the findings of hypoactivation of the orbitofrontal cortex and hyperactivation of the amygdala in many emotional task-based fMRI studies in ADHD [1,28,35–38]. For instance, hyperactivation of the amygdala has been linked to impaired emotional reactivity [28] and delay aversion in reward processing/ choice impulsivity [38] in ADHD. Ventromedial prefrontal cortex (which may include the orbitofrontal cortex) hypoactivation and amygdala hyperactivation in task-based fMRI studies have also been reported in other clinical populations with affect dysregulation [71,72]. Lower amygdalar-ventromedial prefrontal cortex structural connectivity, probed using diffusion tensor imaging measures, has been associated with higher anxiety levels in the healthy population [73]. These task-fMRI studies, among many others, have suggested that an integral role of the amygdala in many emotionally-linked processes of learning, memory, responses and the change of responses [34]. Others have hypothesized that the ventromedial prefrontal cortex is involved in emotional reactivity [74]. Taken together, the findings suggest that the impaired functional integration in task-free affective network, especially the amygdalar and orbitofrontal cortical regions, may underlie or influence the abnormal task-based functional activation during emotion processing in ADHD.

### Limitations and Conclusion

The strengths of our study included a minimum one-month washout period for methylphenidate and fatty acid supplementation (both which could influence the resting-state functional connectivity patterns) [37,75], and a three-month follow-up analysis to determine whether the findings of abnormal intrinsic affective network in ADHD are robust. The primary limitation of our study was the small sample size, which was further reduced by the removal of poor quality data from children who moved excessively during the scan. Low statistical power has been shown to significantly inflate effect sizes of fMRI data [66], hence we cannot make firm conclusions from the brain-behavior correlations found in this study. We urge for further larger-scale studies of the under-investigated affective network in ADHD to be carried out. Additional limitations of our study included missing data on IQ and socioeconomic status. Also, the removal of RS-fMRI frames with excessive motion—though we had limited the removal to less than 10% of the total number—may affect the temporal structure of the data. Further studies to examine whether RS-fMRI measures of affective network is similarly impaired in behaviorally-overlapping disorders such as impulsive-aggressive subtype of conduct disorder [49] and other disorders with severe emotional dysregulation phenotypes such as bipolar disorder [36] could provide additional insight as to whether this surrogate measure could reflect the dimensional construct of emotional disturbance across nosological boundaries [76]. Future work to examine the interaction of the affective network with other networks such as the executive control and salience networks could help elucidate how dsyregulated mood may influence or be influenced by higher order cognitive processes and sensitivity to external salience stimuli.

In conclusion, our study supports the hypothesis that the affective network connectivity is abnormal in children with ADHD. Given that frontolimbic dysfunction continues till adulthood in ADHD [39,77,78] and that emotional dysfunction predicts poor psychosocial long-term outcomes in ADHD [4–8] the role of the intrinsic affective network as a possible biomarker of emotional deficits in larger, multi-site and prospective studies of ADHD should be more thoroughly investigated.

## References

[1] Shaw P, Stringaris A, Nigg J, Leibenluft E (2014) Emotion dysregulation in attention deficit hyperactivity disorder. Am J Psychiatry 171: 276–293. 10.1176/appi.ajp.2013.13070966 24480998 PMC4282137

[2] Barkley RA, Fischer M (2010) The unique contribution of emotional impulsiveness to impairment in major life activities in hyperactive children as adults. J Am Acad Child Adolesc Psychiatry 49: 503–513. 20431470 10.1097/00004583-201005000-00011

[3] Clements SD (1966) The child with minimal brain dysfunction. A multidisciplinary catalyst. J Lancet 86: 121–123. 5904645

[4] Biederman J, Spencer TJ, Petty C, Hyder LL, O'Connor KB, et al (2012) Longitudinal course of deficient emotional self-regulation CBCL profile in youth with ADHD: prospective controlled study. Neuropsychiatr Dis Treat 8: 267–276. 10.2147/NDT.S29670 22848182 PMC3404687

[5] Klein RG, Mannuzza S, Olazagasti MA, Roizen E, Hutchison JA, et al (2012) Clinical and functional outcome of childhood attention-deficit/hyperactivity disorder 33 years later. Arch Gen Psychiatry 69: 1295–1303. 10.1001/archgenpsychiatry.2012.271 23070149 PMC3597443

[6] Wehmeier PM, Schacht A, Barkley RA (2010) Social and emotional impairment in children and adolescents with ADHD and the impact on quality of life. J Adolesc Health 46: 209–217. 10.1016/j.jadohealth.2009.09.009 20159496

[7] Althoff RR, Verhulst FC, Rettew DC, Hudziak JJ, van der Ende J (2010) Adult outcomes of childhood dysregulation: a 14-year follow-up study. J Am Acad Child Adolesc Psychiatry 49: 1105–1116. 10.1016/j.jaac.2010.08.006 20970698 PMC2965164

[8] Surman CB, Biederman J, Spencer T, Miller CA, McDermott KM, et al (2013) Understanding deficient emotional self-regulation in adults with attention deficit hyperactivity disorder: a controlled study. Atten Defic Hyperact Disord 5: 273–281. 10.1007/s12402-012-0100-8 23413201 PMC4009378

[9] Castellanos FX, Proal E (2012) Large-scale brain systems in ADHD: beyond the prefrontal-striatal model. Trends Cogn Sci 16: 17–26. 10.1016/j.tics.2011.11.007 22169776 PMC3272832

[10] Konrad A, Dielentheis TF, El Masri D, Bayerl M, Fehr C, et al (2010) Disturbed structural connectivity is related to inattention and impulsivity in adult attention deficit hyperactivity disorder. Eur J Neurosci 31: 912–919. 10.1111/j.1460-9568.2010.07110.x 20374289

[11] van den Heuvel MP, Hulshoff Pol HE (2010) Exploring the brain network: a review on resting-state fMRI functional connectivity. Eur Neuropsychopharmacol 20: 519–534. 10.1016/j.euroneuro.2010.03.008 20471808

[12] Buckner RL, Krienen FM, Yeo BT (2013) Opportunities and limitations of intrinsic functional connectivity MRI. Nat Neurosci 16: 832–837. 10.1038/nn.3423 23799476

[13] Cole DM, Smith SM, Beckmann CF (2010) Advances and pitfalls in the analysis and interpretation of resting-state FMRI data. Front Syst Neurosci 4: 8 10.3389/fnsys.2010.00008 20407579 PMC2854531

[14] Biswal BB, Mennes M, Zuo XN, Gohel S, Kelly C, et al (2010) Toward discovery science of human brain function. Proc Natl Acad Sci U S A 107: 4734–4739. 10.1073/pnas.0911855107 20176931 PMC2842060

[15] Zuo XN, Kelly C, Adelstein JS, Klein DF, Castellanos FX, et al (2010) Reliable intrinsic connectivity networks: test-retest evaluation using ICA and dual regression approach. Neuroimage 49: 2163–2177. 10.1016/j.neuroimage.2009.10.080 19896537 PMC2877508

[16] Vanderwal T, Kelly C, Castellanos FX (2013) Of bandwagons and bathwater: the value of resting state functional magnetic resonance imaging for child psychiatric research. J Am Acad Child Adolesc Psychiatry 52: 562–565. 10.1016/j.jaac.2013.03.004 23702443

[17] Menon V (2011) Large-scale brain networks and psychopathology: a unifying triple network model. Trends Cogn Sci 15: 483–506. 10.1016/j.tics.2011.08.003 21908230

[18] Uddin LQ, Supekar K, Menon V (2010) Typical and atypical development of functional human brain networks: insights from resting-state FMRI. Front Syst Neurosci 4: 21 10.3389/fnsys.2010.00021 20577585 PMC2889680

[19] Biswal BB, Van Kylen J, Hyde JS (1997) Simultaneous assessment of flow and BOLD signals in resting-state functional connectivity maps. NMR Biomed 10: 165–170. 9430343 10.1002/(sici)1099-1492(199706/08)10:4/5<165::aid-nbm454>3.0.co;2-7

[20] Beckmann CF, DeLuca M, Devlin JT, Smith SM (2005) Investigations into resting-state connectivity using independent component analysis. Philos Trans R Soc Lond B Biol Sci 360: 1001–1013. 16087444 10.1098/rstb.2005.1634 PMC1854918

[21] Choi J, Jeong B, Lee SW, Go HJ (2013) Aberrant development of functional connectivity among resting state-related functional networks in medication-naive ADHD children. PLoS One 8: e83516 10.1371/journal.pone.0083516 24386219 PMC3873390

[22] Hulvershorn LA, Mennes M, Castellanos FX, Di Martino A, Milham MP, et al (2014) Abnormal amygdala functional connectivity associated with emotional lability in children with attention-deficit/hyperactivity disorder. J Am Acad Child Adolesc Psychiatry 53: 351–361 e351. 10.1016/j.jaac.2013.11.012 24565362 PMC3961844

[23] Kucyi A, Hove MJ, Biederman J, Van Dijk KR, Valera EM (2015) Disrupted functional connectivity of cerebellar default network areas in attention-deficit/hyperactivity disorder. Hum Brain Mapp. 10.1002/hbm.22850 PMC4562390 26109476

[24] Li F, He N, Li Y, Chen L, Huang X, et al (2014) Intrinsic brain abnormalities in attention deficit hyperactivity disorder: a resting-state functional MR imaging study. Radiology 272: 514–523. 10.1148/radiol.14131622 24785156

[25] Ray S, Miller M, Karalunas S, Robertson C, Grayson DS, et al (2014) Structural and functional connectivity of the human brain in autism spectrum disorders and attention-deficit/hyperactivity disorder: A rich club-organization study. Hum Brain Mapp 35: 6032–6048. 10.1002/hbm.22603 25116862 PMC4319550

[26] Castellanos FX, Kelly C, Milham MP (2009) The restless brain: attention-deficit hyperactivity disorder, resting-state functional connectivity, and intrasubject variability. Can J Psychiatry 54: 665–672. 19835673 10.1177/070674370905401003 PMC3876940

[27] Tomasi D, Volkow ND (2012) Abnormal functional connectivity in children with attention-deficit/hyperactivity disorder. Biol Psychiatry 71: 443–450. 10.1016/j.biopsych.2011.11.003 22153589 PMC3479644

[28] Posner J, Nagel BJ, Maia TV, Mechling A, Oh M, et al (2011) Abnormal amygdalar activation and connectivity in adolescents with attention-deficit/hyperactivity disorder. J Am Acad Child Adolesc Psychiatry 50: 828–837 e823. 10.1016/j.jaac.2011.05.010 21784302 PMC3155780

[29] Uddin LQ, Kelly AM, Biswal BB, Margulies DS, Shehzad Z, et al (2008) Network homogeneity reveals decreased integrity of default-mode network in ADHD. J Neurosci Methods 169: 249–254. 10.1016/j.jneumeth.2007.11.031 18190970

[30] LeDoux JE (2000) Emotion circuits in the brain. Annu Rev Neurosci 23: 155–184. 10845062 10.1146/annurev.neuro.23.1.155

[31] Maclean PD (1955) The limbic system ("visceral brain") and emotional behavior. AMA Arch Neurol Psychiatry 73: 130–134. 13227663 10.1001/archneurpsyc.1955.02330080008004

[32] Papez JW (1995) A proposed mechanism of emotion. 1937. J Neuropsychiatry Clin Neurosci 7: 103–112. 7711480 10.1176/jnp.7.1.103

[33] Dolan RJ (2007) The human amygdala and orbital prefrontal cortex in behavioural regulation. Philos Trans R Soc Lond B Biol Sci 362: 787–799. 17403643 10.1098/rstb.2007.2088 PMC2429997

[34] Phelps EA (2006) Emotion and cognition: insights from studies of the human amygdala. Annu Rev Psychol 57: 27–53. 16318588 10.1146/annurev.psych.56.091103.070234

[35] Strohle A, Stoy M, Wrase J, Schwarzer S, Schlagenhauf F, et al (2008) Reward anticipation and outcomes in adult males with attention-deficit/hyperactivity disorder. Neuroimage 39: 966–972. 17996464 10.1016/j.neuroimage.2007.09.044

[36] Brotman MA, Rich BA, Guyer AE, Lunsford JR, Horsey SE, et al (2010) Amygdala activation during emotion processing of neutral faces in children with severe mood dysregulation versus ADHD or bipolar disorder. Am J Psychiatry 167: 61–69. 10.1176/appi.ajp.2009.09010043 19917597 PMC3075433

[37] Rubia K, Halari R, Cubillo A, Mohammad AM, Brammer M, et al (2009) Methylphenidate normalises activation and functional connectivity deficits in attention and motivation networks in medication-naive children with ADHD during a rewarded continuous performance task. Neuropharmacology 57: 640–652. 10.1016/j.neuropharm.2009.08.013 19715709

[38] Plichta MM, Vasic N, Wolf RC, Lesch KP, Brummer D, et al (2009) Neural hyporesponsiveness and hyperresponsiveness during immediate and delayed reward processing in adult attention-deficit/hyperactivity disorder. Biol Psychiatry 65: 7–14. 10.1016/j.biopsych.2008.07.008 18718573

[39] McCarthy H, Skokauskas N, Mulligan A, Donohoe G, Mullins D, et al (2013) Attention network hypoconnectivity with default and affective network hyperconnectivity in adults diagnosed with attention-deficit/hyperactivity disorder in childhood. JAMA Psychiatry 70: 1329–1337. 10.1001/jamapsychiatry.2013.2174 24132732

[40] Posner J, Rauh V, Gruber A, Gat I, Wang Z, et al (2013) Dissociable attentional and affective circuits in medication-naive children with attention-deficit/hyperactivity disorder. Psychiatry Res 213: 24–30. 10.1016/j.pscychresns.2013.01.004 23664625 PMC3717483

[41] Yeo BT, Krienen FM, Sepulcre J, Sabuncu MR, Lashkari D, et al (2011) The organization of the human cerebral cortex estimated by intrinsic functional connectivity. J Neurophysiol 106: 1125–1165. 10.1152/jn.00338.2011 21653723 PMC3174820

[42] Wimberger HC, Gregory RJ (1968) A behavior checklist for use in child psychiatry clinics. J Am Acad Child Psychiatry 7: 677–688. 5724551 10.1016/s0002-7138(09)62187-7

[43] Shaffer D, Gould MS, Brasic J, Ambrosini P, Fisher P, et al (1983) A children's global assessment scale (CGAS). Arch Gen Psychiatry 40: 1228–1231. 6639293 10.1001/archpsyc.1983.01790100074010

[44] Achenbach TM, A. R L (2014) ASEBA Child Behavior Checklist for Ages 6–18 (CBCL/6-18).

[45] Achenbach TM, Dumenci L, Rescorla LA (2003) DSM-oriented and empirically based approaches to constructing scales from the same item pools. J Clin Child Adolesc Psychol 32: 328–340. 12881022 10.1207/S15374424JCCP3203_02

[46] Cha J, Fekete T, Siciliano F, Biezonski D, Greenhill L, et al (2015) Neural Correlates of Aggression in Medication-Naive Children with ADHD: Multivariate Analysis of Morphometry and Tractography. Neuropsychopharmacology 40: 1717–1725. 10.1038/npp.2015.18 25645374 PMC4915254

[47] Gotham K, Brunwasser SM, Lord C (2015) Depressive and anxiety symptom trajectories from school age through young adulthood in samples with autism spectrum disorder and developmental delay. J Am Acad Child Adolesc Psychiatry 54: 369–376 e363. 10.1016/j.jaac.2015.02.005 25901773 PMC4407021

[48] Mazzone L, Postorino V, De Peppo L, Fatta L, Lucarelli V, et al (2013) Mood symptoms in children and adolescents with autism spectrum disorders. Res Dev Disabil 34: 3699–3708. 10.1016/j.ridd.2013.07.034 24029798

[49] Rubia K (2011) "Cool" inferior frontostriatal dysfunction in attention-deficit/hyperactivity disorder versus "hot" ventromedial orbitofrontal-limbic dysfunction in conduct disorder: a review. Biol Psychiatry 69: e69–87. 10.1016/j.biopsych.2010.09.023 21094938

[50] Spencer TJ, Faraone SV, Surman CB, Petty C, Clarke A, et al (2011) Toward defining deficient emotional self-regulation in children with attention-deficit/hyperactivity disorder using the Child Behavior Checklist: a controlled study. Postgrad Med 123: 50–59. 10.3810/pgm.2011.09.2459 21904086 PMC3737570

[51] Zuo XN, Ehmke R, Mennes M, Imperati D, Castellanos FX, et al (2012) Network centrality in the human functional connectome. Cereb Cortex 22: 1862–1875. 10.1093/cercor/bhr269 21968567

[52] Jenkinson M, Beckmann CF, Behrens TE, Woolrich MW, Smith SM (2012) Fsl. Neuroimage 62: 782–790. 10.1016/j.neuroimage.2011.09.015 21979382

[53] Cox RW (1996) AFNI: software for analysis and visualization of functional magnetic resonance neuroimages. Comput Biomed Res 29: 162–173. 8812068 10.1006/cbmr.1996.0014

[54] Greve DN, Fischl B (2009) Accurate and robust brain image alignment using boundary-based registration. Neuroimage 48: 63–72. 10.1016/j.neuroimage.2009.06.060 19573611 PMC2733527

[55] Power JD, Barnes KA, Snyder AZ, Schlaggar BL, Petersen SE (2012) Spurious but systematic correlations in functional connectivity MRI networks arise from subject motion. Neuroimage 59: 2142–2154. 10.1016/j.neuroimage.2011.10.018 22019881 PMC3254728

[56] Van Dijk KR, Sabuncu MR, Buckner RL (2012) The influence of head motion on intrinsic functional connectivity MRI. Neuroimage 59: 431–438. 10.1016/j.neuroimage.2011.07.044 21810475 PMC3683830

[57] Ashburner J, Chen C-C, Daunizeau J, Flandin G, Friston K, Gitelman D, et al. (July 2010) SPM8 manual.

[58] Ashburner J, Friston KJ (2000) Voxel-based morphometry—the methods. Neuroimage 11: 805–821. 10860804 10.1006/nimg.2000.0582

[59] Jafri MJ, Pearlson GD, Stevens M, Calhoun VD (2008) A method for functional network connectivity among spatially independent resting-state components in schizophrenia. Neuroimage 39: 1666–1681. 18082428 10.1016/j.neuroimage.2007.11.001 PMC3164840

[60] Calhoun VD, Adali T, Pearlson GD, Pekar JJ (2001) A method for making group inferences from functional MRI data using independent component analysis. Hum Brain Mapp 14: 140–151. 11559959 10.1002/hbm.1048 PMC6871952

[61] Erhardt EB, Rachakonda S, Bedrick EJ, Allen EA, Adali T, et al (2011) Comparison of multi-subject ICA methods for analysis of fMRI data. Hum Brain Mapp 32: 2075–2095. 10.1002/hbm.21170 21162045 PMC3117074

[62] Ashburner J, Chen C-C, Daunizeau J, Flandin G, Friston K, Gitelman D, et al. (July 2010) SPM8 manual.

[63] Zhou J, Greicius MD, Gennatas ED, Growdon ME, Jang JY, et al (2010) Divergent network connectivity changes in behavioural variant frontotemporal dementia and Alzheimer's disease. Brain 133: 1352–1367. 10.1093/brain/awq075 20410145 PMC2912696

[64] Poline JB, Worsley KJ, Evans AC, Friston KJ (1997) Combining spatial extent and peak intensity to test for activations in functional imaging. Neuroimage 5: 83–96. 9345540 10.1006/nimg.1996.0248

[65] Casanova R, Srikanth R, Baer A, Laurienti PJ, Burdette JH, et al (2007) Biological parametric mapping: A statistical toolbox for multimodality brain image analysis. Neuroimage 34: 137–143. 17070709 10.1016/j.neuroimage.2006.09.011 PMC1994117

[66] Yarkoni T (2009) Big Correlations in Little Studies: Inflated fMRI Correlations Reflect Low Statistical Power-Commentary on Vul et al (2009). Perspect Psychol Sci 4: 294–298. 10.1111/j.1745-6924.2009.01127.x 26158966

[67] Hesslinger B, Tebartz van Elst L, Thiel T, Haegele K, Hennig J, et al (2002) Frontoorbital volume reductions in adult patients with attention deficit hyperactivity disorder. Neurosci Lett 328: 319–321. 12147334 10.1016/s0304-3940(02)00554-2

[68] Plessen KJ, Bansal R, Zhu H, Whiteman R, Amat J, et al (2006) Hippocampus and amygdala morphology in attention-deficit/hyperactivity disorder. Arch Gen Psychiatry 63: 795–807. 16818869 10.1001/archpsyc.63.7.795 PMC2367150

[69] Vincent JL, Patel GH, Fox MD, Snyder AZ, Baker JT, et al (2007) Intrinsic functional architecture in the anaesthetized monkey brain. Nature 447: 83–86. 17476267 10.1038/nature05758

[70] Cortese S, Imperati D, Zhou J, Proal E, Klein RG, et al (2013) White matter alterations at 33-year follow-up in adults with childhood attention-deficit/hyperactivity disorder. Biol Psychiatry 74: 591–598. 10.1016/j.biopsych.2013.02.025 23566821 PMC3720804

[71] Etkin A, Wager TD (2007) Functional neuroimaging of anxiety: a meta-analysis of emotional processing in PTSD, social anxiety disorder, and specific phobia. Am J Psychiatry 164: 1476–1488. 17898336 10.1176/appi.ajp.2007.07030504 PMC3318959

[72] Shin LM, Wright CI, Cannistraro PA, Wedig MM, McMullin K, et al (2005) A functional magnetic resonance imaging study of amygdala and medial prefrontal cortex responses to overtly presented fearful faces in posttraumatic stress disorder. Arch Gen Psychiatry 62: 273–281. 15753240 10.1001/archpsyc.62.3.273

[73] Kim MJ, Whalen PJ (2009) The structural integrity of an amygdala-prefrontal pathway predicts trait anxiety. J Neurosci 29: 11614–11618. 10.1523/JNEUROSCI.2335-09.2009 19759308 PMC2791525

[74] Anderson SW, Barrash J, Bechara A, Tranel D (2006) Impairments of emotion and real-world complex behavior following childhood- or adult-onset damage to ventromedial prefrontal cortex. J Int Neuropsychol Soc 12: 224–235. 16573856 10.1017/S1355617706060346

[75] Mueller S, Costa A, Keeser D, Pogarell O, Berman A, et al (2014) The effects of methylphenidate on whole brain intrinsic functional connectivity. Hum Brain Mapp 35: 5379–5388. 10.1002/hbm.22557 24862742 PMC6869774

[76] Chabernaud C, Mennes M, Kelly C, Nooner K, Di Martino A, et al (2012) Dimensional brain-behavior relationships in children with attention-deficit/hyperactivity disorder. Biol Psychiatry 71: 434–442. 10.1016/j.biopsych.2011.08.013 21974788 PMC3568534

[77] Schulz KP, Bedard AC, Fan J, Clerkin SM, Dima D, et al (2014) Emotional bias of cognitive control in adults with childhood attention-deficit/hyperactivity disorder. Neuroimage Clin 5: 1–9. 10.1016/j.nicl.2014.05.016 24918067 PMC4050315

[78] Cocchi L, Bramati IE, Zalesky A, Furukawa E, Fontenelle LF, et al (2012) Altered functional brain connectivity in a non-clinical sample of young adults with attention-deficit/hyperactivity disorder. J Neurosci 32: 17753–17761. 10.1523/JNEUROSCI.3272-12.2012 23223295 PMC6621678

